# One‐Electron Oxidation of [M(P^t^Bu_3_)_2_] (M=Pd, Pt): Isolation of Monomeric [Pd(P^t^Bu_3_)_2_]^+^ and Redox‐Promoted C−H Bond Cyclometalation

**DOI:** 10.1002/anie.201511467

**Published:** 2016-02-16

**Authors:** Thibault Troadec, Sze‐yin Tan, Christopher J. Wedge, Jonathan P. Rourke, Patrick R. Unwin, Adrian B. Chaplin

**Affiliations:** ^1^ Department of ChemistryUniversity of WarwickGibbet Hill RoadCoventryCV4 7ALUK; ^2^ Department of PhysicsUniversity of WarwickGibbet Hill RoadCoventryCV4 7ALUK

**Keywords:** C−H activation, oxidation, palladium, phosphane ligands, platinum

## Abstract

Oxidation of zero‐valent phosphine complexes [M(P^t^Bu_3_)_2_] (M=Pd, Pt) has been investigated in 1,2‐difluorobenzene solution using cyclic voltammetry and subsequently using the ferrocenium cation as a chemical redox agent. In the case of palladium, a mononuclear paramagnetic Pd^I^ derivative was readily isolated from solution and fully characterized (EPR, X‐ray crystallography). While in situ electrochemical measurements are consistent with initial one‐electron oxidation, the heavier congener undergoes C−H bond cyclometalation and ultimately affords the 14 valence‐electron Pt^II^ complex [Pt(*κ* ^2^_PC_‐P^t^Bu_2_CMe_2_CH_2_)(P^t^Bu_3_)]^+^ with concomitant formation of [Pt(P^t^Bu_3_)_2_H]^+^.

Over the past few decades a rich variety of chemistry has emerged based on the reactions of palladium and platinum complexes in the 0 and +II formal oxidation states, epitomized by the omnipresence of palladium catalyzed cross‐coupling reactions in contemporary organic chemistry.^1^, ^2^ In contrast, the organometallic chemistry of well‐defined complexes of these elements bearing formal +I oxidation states is much less established and examples are largely limited to unstable or dinuclear species with distinct metal–metal bonds.^3^, ^4^ Halogen bridged palladium complexes of the type [Pd(*μ*‐X)(P^t^Bu_3_)]_2_ (X=Br, I) are notable examples and are believed to act as reservoirs for reactive {Pd^0^(P^t^Bu_3_)} fragments in catalytic transformations.^5^ In other systems, Pd^I^ and Pt^I^ species have been postulated as intermediates, but with little supporting evidence.^6^ With a view to isolating well‐defined mononuclear complexes in the +I oxidation state relevant to catalysis, we report herein our work involving one‐electron oxidation of widely used and commercially available palladium(0) and platinum(0) complexes of tri‐*tert*‐butylphosphine [M^0^(P^t^Bu_3_)_2_] (M=Pd, **1 a**; Pt, **1 b**).

As a starting point we determined the redox potentials of **1 a** and **1 b** by cyclic voltammetry (CV) in the weakly coordinating solvent 1,2‐difluorobenzene (0.2 m [^n^Bu_4_N][PF_6_] electrolyte, Figure 1).^7^ Reversible one‐electron oxidation was observed at *E*_1/2_=−0.44 V (**1 a**) and *E*_1/2_=−0.10 V (**1 b**) relative to Fc/[Fc]^+^ (Fc=ferrocene). The electrochemical characteristics of closely related cyclic alkyl(amino) carbene (CAAC) analogues have recently been studied by CV and the redox potentials of **1 a** and **1 b** are similar in magnitude to those found for [M^0^(CAAC)_2_] (M=Pd, −0.60 V; Pt, −0.07 V) in THF (0.1 m [^n^Bu_4_N][ClO_4_]).^8^ Consistent with the generation of a stable Pd^I^ species (**2 a**), the peak current ratios (*i*_p_^red^/*i*_p_^ox^) in the palladium voltammograms are essentially unity (ca. 0.99). Conspicuously lower ratios were observed for the platinum complex (ca. 0.90).

**Figure 1:**
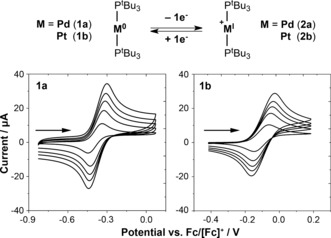
Cyclic voltammograms for the oxidation of **1 a** and **1 b** in 1,2‐C_6_H_4_F_2_ (2 mm **1**; 0.2 m [^n^Bu_4_N][PF_6_] electrolyte; glassy carbon working electrode, Pt counter electrode and Ag wire reference electrode; scan rates=10, 30, 50, 70, and 100 mV s^−1^).

Encouraged by these data, **1 a** was reacted with one equiv of [Fc][PF_6_] in 1,2‐difluorobenzene at 293 K and dark blue [Pd^I^(P^t^Bu_3_)_2_][PF_6_] **2 a** was subsequently isolated in 92 % yield following addition of *n*‐pentane. The electrochemical characteristics of isolated **2 a** are equivalent to those measured in situ starting from **1 a** (*E*_1/2_=−0.42 V; see Supporting Information). This new paramagnetic species was additionally characterized in solution using UV/Vis spectroscopy (*λ*_max_=667 nm), ESI‐HRMS (positive ion mode, 510.2736 *m*/*z* [*M*]^+^; calculated 510.2740 *m*/*z*), and EPR spectroscopy. The EPR spectrum (1,2‐C_6_H_4_F_2_ glass at 200 K, Figure 2), shows a superposition of a single resonance at *g=*2.316(5) with a lower intensity sextet arising from hyperfine coupling to ^105^Pd (*I*=5/2, 22 % abundance), corroborating formation of an *S=*1/2 Pd^I^ species. The unusually large ^105^Pd hyperfine coupling of approximately 25 mT, and lack of resolved coupling to ^31^P (*I*=1/2, 100 % abundance) is consistent with strong localization of the unpaired electron spin on the Pd center. Complex **2 a** crystallizes in the high‐symmetry cubic space group *Pa* $\bar{3}$ with the palladium atom on a center of inversion (Figure 2). In comparison to **1 a**, the Pd−P bond length is significantly elongated, from 2.285(3) to 2.3469(6) Å (Δ(Pd−P)=+0.062(4) Å); the P‐Pd‐P angles in both cases are symmetry enforced at 180°.^9^ To the best of our knowledge, this is the first example of an unsupported two‐coordinate Pd^I^ complex. A similar bond length elongation has been noted in closely related NHC complexes of Ni^0^/Ni^I^ (Δ(Ni−C)=+0.08(2) Å).^10^ Isolated **2 a** is air‐sensitive in solution, but shows good stability under an argon atmosphere. For instance, under argon the EPR spectrum intensity was essentially unchanged after 24 h at 293 K (15 mm). However, slow degradation of **2 a** was observed by UV/Vis spectroscopy under high dilution conditions (*t*_1/2_≈30 h; 0.15 mm), which we attribute to the presence of adventitious water as the rate of degradation increased significantly when water was added deliberately. Moreover, **2 a** can be stored in the solid‐state in air (72 h) with no evident change by UV/Vis spectroscopy.

**Figure 2:**
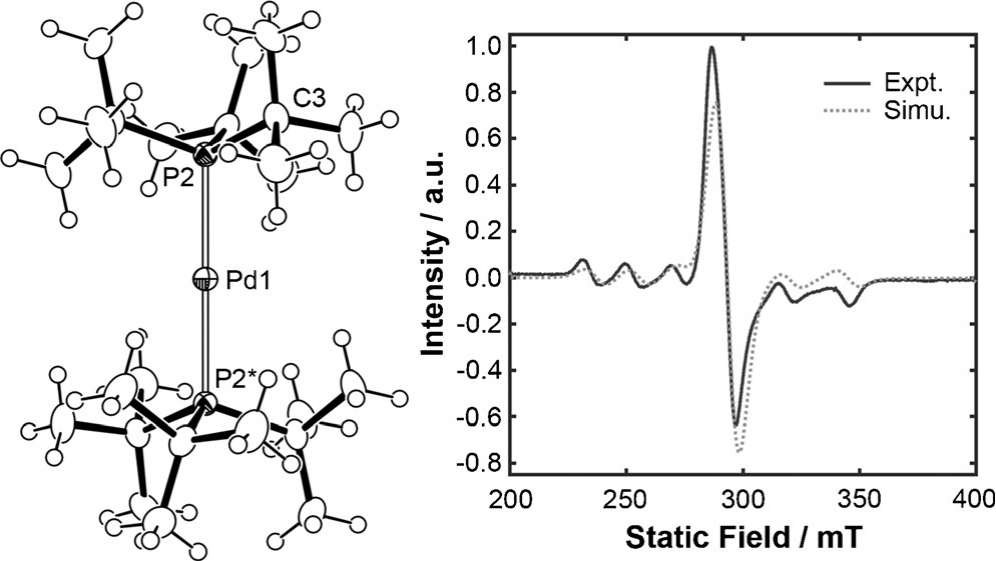
The solid‐state structure^22^ and EPR spectrum of **2 a** (1,2‐C_6_H_4_F_2_ glass, 200 K, a.u.=arbitrary units).^11^ Ellipsoids are set at 50 % probability; anion omitted for clarity. The starred atom is generated by the symmetry operation 1−*x*, 1−*y*, 1−*z*. Selected data: Pd1−P2 2.3470(6) Å; P2‐Pd1‐P2* 180°, Pd1‐P2‐C3 108.81(5)°.

When preparation of the analogous Pt^I^ complex **2 b** was attempted by reaction of **1 b** with one equiv of [Fc][PF_6_], a 1:1 mixture of the new diamagnetic cyclometalated complex [Pt^II^(*κ* ^2^_PC_‐P^t^Bu_2_CMe_2_CH_2_)(P^t^Bu_3_)][PF_6_] **3 b** and known Pt^II^ hydride [Pt^II^(P^t^Bu_3_)_2_H][PF_6_] **4** (*δ*(^1^H) −36.30 ppm; ^2^ *J*_PH_=8.6, ^1^ *J*_PtH_=2590 Hz; *δ*(^31^P) 86.3 ppm; ^1^ *J*_PtP_=2621 Hz) was formed within 15 min instead, as indicated by ^1^H and ^31^P NMR spectroscopy (Fc observed; Scheme 1).^12^ This outcome suggests only transient stability of **2 b** in solution, with subsequent C−H bond homolysis accounting for the divergence from fully reversible one‐electron oxidation of **1 b** observed by CV.^13^ Reaction of **1 b** with two equiv of [Fc][PF_6_] in the presence of excess hindered base 2,6‐bis(decyl)pyridine (5 equiv), which is able to deprotonate **4**, resulted in selective formation of **3 b** within 15 min. In this manner, **3 b** was isolated in 93 % yield following successive crystallizations from 1,2‐C_6_H_4_F_2_ to remove ferrocene, excess base, and pyridinium salt.^13^ For comparison, no significant reaction was detected by ^1^H or ^31^P NMR spectroscopy on mixing of **1 b** and 2,6‐bis(decyl)pyridine in 1,2‐difluorobenzene at 293 K (24 h) or heating **1 b** alone in 1,2‐difluorobenzene at 353 K (24 h).

**Scheme 1:**
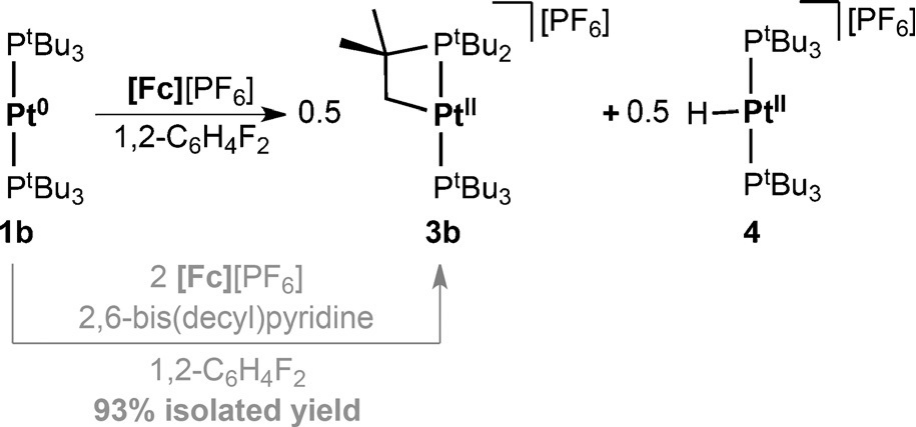
Chemical oxidation of **1 b**.

Two independent but structurally similar cations are observed in the solid‐state structure of **3 b** (one is shown in Figure 3), both illustrating adoption of a T‐shaped coordination geometry^14^ and cyclometalation of one of the *tert*‐butyl substituents; these are identified by distinctly acute Pt1‐P2‐C3 angles [90.0(3)/89.5(3)°] and Pt1−C4 bond lengths of 2.063(17)/2.065(17) Å. The **3 b** cation is formally a 14 valence‐electron (VE) complex, but is stabilized by adoption of an agostic interaction between the non‐cyclometalated phosphine ligand and Pt center (Pt1⋅⋅⋅C4B 2.83(2)/2.84(2) Å). In solution, the structure of **3 b** was fully corroborated by NMR spectroscopy (CD_2_Cl_2_, 298 K). Formation of the metallacycle is apparent by distinctive ^1^H and ^13^C methylene resonances at *δ*(^1^H) 2.75 ppm (^2^ *J*_PtH_=110 Hz) and *δ*(^13^C) 10.3 ppm (^1^ *J*_PtC_=670 Hz) with platinum satellites, two doublet ^31^P resonances with a large (*trans*) ^2^ *J*_PP_ coupling constant and platinum satellites (*δ*(^31^P) 59.1 ppm (^1^ *J*_PtP_=2896 Hz, ^2^ *J*_PP_=317 Hz, P ^t^Bu_3_), *δ*(^31^P) 25.2 ppm (^1^ *J*_PtP_=1916 Hz, ^2^ *J*_PP_=317 Hz, P ^t^Bu_2_CMe_2_CH_2_)), and a platinum chemical shift of *δ*(^195^Pt) −3816 ppm (225 K). Although the signals associated with the non‐cyclometalated phosphine ligand broadened on cooling to 185 K, the agostic interaction could not be definitively resolved by ^1^H NMR spectroscopy.

**Figure 3:**
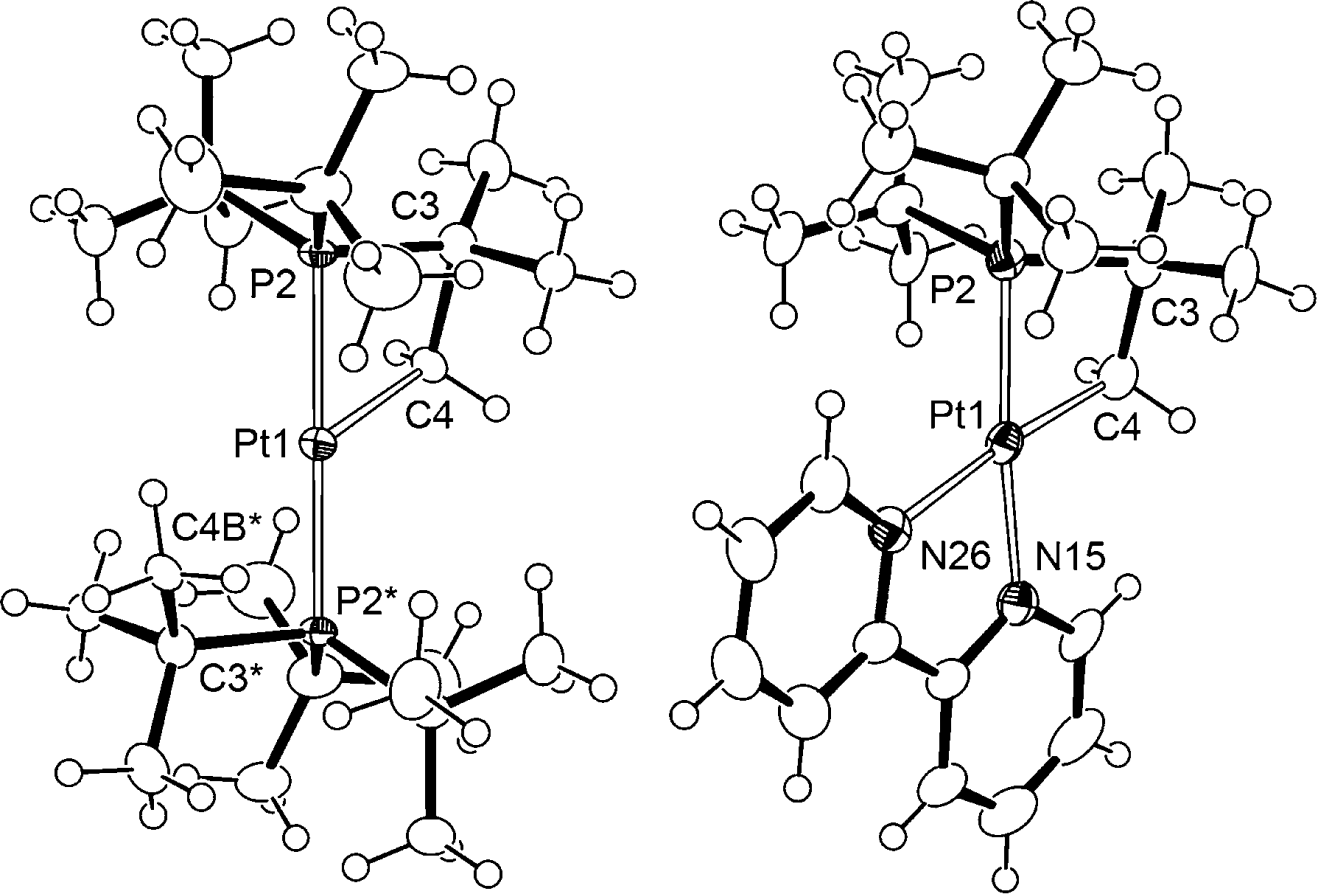
Solid‐state structures of **3 b** and **6**.^22^ Ellipsoids are set at 50 % and 30 % probability, respectively; minor disordered components and anions omitted for clarity; only one of the two independent molecules is shown for **3 b**. Starred atoms in **3 b** are generated by the symmetry operation 1−*x*, 2−*y*, 1−*z*. Selected data **3 b**: Pt1−P2 2.297(2) Å, Pt1−C4 2.063(17) Å, Pt1⋅⋅⋅C4B* 2.83(2) Å; P2‐Pt1‐P2* 180°, Pt1‐P2‐C3/C3* 90.0(3)°. **6**: Pt1−P2 2.235(2) Å, Pt1−C4 2.077(10) Å, Pt1−N15 2.080(7) Å, Pt1−N26 2.156(7) Å; P2‐Pt1‐N15 166.3(2)°, C4‐Pt1‐N26 175.4(3)°, Pt1‐P2‐C3 88.5(3)°.

Cyclometalation reactions of Pt^II^ complexes have extensive precedent.14a, 15 For instance, T‐shaped complexes [Pt^II^(*κ* ^2^_PC_‐PR_2_C_6_H_3_MeCH_2_)(PR_2_Xyl)]^+^ (R=Cy, Ph; Xyl = 2,6‐dimethylphenyl) with similar structural and spectroscopic metrics compared to **3 b**, were prepared by cyclometalation reactions involving halide abstraction from [Pt^II^(PR_2_Xyl)_2_(Me)Cl] and subsequent elimination of methane.^16^ Intramolecular C−H bond activation of P^t^Bu_3_ in [Pt^II^(P^t^Bu_3_)_2_HX] (X=Cl, Br, I, OTf, NO_2_) has also been described and results in coordinatively saturated products [Pt^II^(*κ* ^2^_PC_‐P^t^Bu_2_CMe_2_CH_2_)(P^t^Bu_3_)X].^17^ In the case of **3 b**, the presence of a Pt^II^ intermediate proceeding cyclometalation can be discounted on the basis of the electrochemical characteristics of **1 b**. Instead the formation of **3 b** and **4** presumably occurs via concerted bimetallic (radical) oxidative addition,^18^ or proceeds through a common Pt^III^ alkyl hydride intermediate [Pt^II^(*κ* ^2^_PC_‐P^t^Bu_2_CMe_2_CH_2_)(P^t^Bu_3_)H]^+^ (**5**). In the latter case, subsequent comproportionation (**5**+**2 b**), disproportion (via a Pt^IV^ alkyl dihydride), or Pt−H bond homolysis (i.e. 2×**5**→2×**3 b**+H_2_; **3 b**+H_2_→**4**) would afford the observed 1:1 mixture of **3 b** and **4**.^19^

Seeking to gain more insight into this mechanism, trapping of the postulated intermediate **5** was attempted by coordination of 2,2′‐bipyridine (bipy). However, oxidation of **1 b** with either one or two equiv of [Fc][PF_6_] in the presence of one equiv of bipy resulted in formation of a new cyclometalated complex [Pt^II^(*κ* ^2^_PC_‐P^t^Bu_2_CMe_2_CH_2_)(bipy)][PF_6_] **6** instead, alongside protonated phosphine (δ(^31^P) 54.2 ppm). The identity of this new complex was verified by independent synthesis from **3 b** and bipy in 1,2‐C_6_H_4_F_2_ (97 % yield of isolated product). As with **3 b**, the cyclometalated phosphine in **6** is characterized by an acute Pt1‐P2‐C3 angle (88.5(3)°) and bears a similar Pt1−C4 bond length of 2.077(10) Å (Figure 3). Moreover, both solution and solid‐state data are fully consistent with a coordinatively saturated metal complex. Notably, the substantially higher *trans*‐influence of the methylene ligand is reflected in different Pt−N bond lengths (Pt1−N15, 2.156(7) versus Pt1−N26, 2.080(7) Å); the associated ^13^C resonance shows a reduced ^1^ *J*_PtC_ coupling in comparison to **3 b** (580 versus 670 Hz). Stronger Pt−P bonding is apparent in **6** relative to **3 b**, on the basis of a shorter Pt−P bond (2.235(2) versus 2.297(2)/2.299(3) Å), and a larger ^1^ *J*_PtP_ coupling constant determined by ^31^P NMR spectroscopy (3105 versus 1916 Hz). A platinum chemical shift of *δ*(^195^Pt) −3788 ppm (225 K) was also measured for **6** and is very similar to that of **3 b** (*δ*(^195^Pt) −3816 ppm).

Reaction of isolated **3 b** with H_2_ (1 atm) results directly in the formation of **4**, which is reconcilable with Pt−H bond homolysis or disproportion (via an unstable Pt^IV^ alkyl dihydride intermediate) during the formation of **3 b**/**4**. However, the underlying mechanism is still not completely clear at this time. For instance, we cannot discount the formation of **3 b** through a pathway involving deprotonation of **5** (mediated by **1 b** 20 or 2,6‐bis(decyl)pyridine) and a second one‐electron oxidation. The redox potential of the associated Pt^I^/Pt^II^ couple, assessed by CV experiments using both isolated **3 b** (*E*_1/2_=−1.90 V, irreversible) and **6** (*E*_1/2_=−1.68 V, *i*_p_^ox^/*i*_p_^red^≈0.96), indicates that such a one‐electron oxidation is at least conceptually feasible using [Fc][PF_6_] (see Supporting Information for CVs).

Motivated by the cyclometalation observed on oxidation of **1 b**, we have also preliminarily investigated whether similar reactivity can be induced in the palladium analogue. Our studies are on‐going, but we do note that reaction of **1 a** with two equiv of [Fc][PF_6_] in the presence of excess 2,6‐bis(decyl)pyridine (5 equiv) resulted in the gradual appearance of a diamagnetic complex with spectroscopic characteristics consistent with cyclometalation (**3 a**; *δ*(^31^P) 57.0, −1.3 ppm; ^2^ *J*_PP_=316 Hz).^21^ However, this species was only formed in situ in about 30 % yield after 72 h at 293 K, as measured by NMR spectroscopy (using an internal standard), and the resulting reaction mixture has proved intractable so far to further characterization.

In summary, we have described a simple method for accessing the reaction chemistry of mononuclear palladium and platinum complexes bearing a +I formal oxidation state, as demonstrated by one‐electron oxidation of [M^0^(P^t^Bu_3_)_2_] (M=Pd, Pt) using [Fc][PF_6_]. While the Pd^I^ derivative was readily isolated from solution and fully characterized, the heavier congener undergoes C−H bond cyclometalation to afford the 14 VE Pt^II^ complex [Pt^II^(*κ* ^2^_PC_‐P^t^Bu_2_CMe_2_CH_2_)(P^t^Bu_3_)]^+^ with concomitant formation of [Pt^II^(P^t^Bu_3_)_2_H]^+^. Future work is focused on charting the reactivity and catalytic activity of these novel Group 10 species, and will be published in due course.

## Supporting information

As a service to our authors and readers, this journal provides supporting information supplied by the authors. Such materials are peer reviewed and may be re‐organized for online delivery, but are not copy‐edited or typeset. Technical support issues arising from supporting information (other than missing files) should be addressed to the authors.

Supplementary
