# Supplementary material for: One‐Electron Oxidation of [M(PtBu3)2] (M=Pd, Pt): Isolation of Monomeric [Pd(PtBu3)2]+ and Redox‐Promoted C−H Bond Cyclometalation
Source: Angew Chem Int Ed Engl. 2016 Feb 16;55(11):3754–7. doi: 10.1002/anie.201511467 (PMC4797707; doi:10.1002/anie.201511467)
Supplement: Supplementary file 1 — Supplementary [file ANIE-55-3754-s001.pdf]

Angewandte Chemie
Eine Zeitschrift der Gesellschaft Deutscher Chemiker

Supporting Information

One-Electron Oxidation of $[M(P^tBu_3)_2]$ (M = Pd, Pt): Isolation of Monomeric $[Pd(P^tBu_3)_2]^+$ and Redox-Promoted C–H Bond Cyclometalation

*Thibault Troadec, Sze-yin Tan, Christopher J. Wedge, Jonathan P. Rourke, Patrick R. Unwin, and Adrian B. Chaplin**

anie_201511467_sm_miscellaneous_information.pdf

Contents

1 Synthesis of new compounds **2**

1.1 General methods *2*

*1.2 Preparation of [Pd(P^{t}Bu$_3$)$_2$][PF$_6$] (**2a**)* *3*

1.3 Preparation of 2,6-bis(decyl)pyridine *4*

*1.4 Preparation of [Pt(κ$^2_{PC}$–P^{t}Bu$_2$CMe$_2$CH$_2$)(P^{t}Bu$_3$)][PF$_6$] (**3b**)* *4*

*1.5 Preparation of [Pt(κ$^2_{PC}$–P^{t}Bu$_2$CMe$_2$CH$_2$)(bipy)][PF$_6$] (**6**)* *6*

2 NMR scale reaction details **8**

2.1 ^{31}P Chemical shifts in 1,2-C$_6$H$_4$F$_2$ *8*

2.2 General conditions *8*

*2.3 Reactions of [Pd(P^{t}Bu$_3$)$_2$] (**1a**)* *8*

*2.4 Reactions of [Pt(P^{t}Bu$_3$)$_2$] (**1b**)* *9*

*2.5 Reactions of [Pd(P^{t}Bu$_3$)$_2$][PF$_6$] (**2a**)* *10*

*2.6 Reactions of [Pt(κ$^2_{PC}$–P^{t}Bu$_2$CMe$_2$CH$_2$)(P^{t}Bu$_3$)][PF$_6$] (**3b**)* *10*

3 Electrochemistry **11**

3.1 General methods *11*

3.2 Fc/[Fc]$^+$ redox couple in 1,2-C$_6$H$_4$F$_2$ / 0.2 M [^{n}Bu$_4$N][PF$_6$] *11*

*3.3 Reduction of isolated **2a*** *12*

*3.4 Reduction of isolated **3b** and **6*** *12*

3.5 Additional details *12*

4 EPR Spectroscopy **14**

5 Crystallography **14**

6 References and notes **15**

1 Synthesis of new compounds

1.1 General methods

All manipulations were performed under an atmosphere of argon, using Schlenk and glove box techniques. Glassware was oven dried at 150ºC overnight and flamed under vacuum prior to use. Anhydrous CH_2Cl_2, THF and pentane (<0.005% H_2O) were purchased from ACROS or Sigma-Aldrich and freeze-pump-thaw degassed three times before being placed under argon. CD_2Cl_2 was dried over CaH_2, vacuum distilled, and freeze-pump-thaw degassed three times before being placed under argon. 1,2-Difluorobenzene (DiFB) was stirred over neutral aluminum oxide, filtered, dried over CaH_2, vacuum distilled, and freeze-pump-thaw degassed three times before being placed under argon over 3 Å molecular sieves. [Pd(P^tBu_3)$_2$] (**1a**), [Pt(P^tBu_3)$_2$] (**1b**) and [Fc][PF_6] were purchased from Sigma-Aldrich and used as received. Na[BAr^F_4] was synthesised using a literature protocol. [1]

[Fc][BAr^F_4] was prepared using an adapted literature procedure.[2] A suspension of [Fc][PF_6] (120 mg, 0.362 mmol) and Na[BAr^F_4] (354 mg, 0.398 mmol) in dry CH_2Cl_2 (15 mL) was stirred at room temperature for 3 h. The solution was filtered and layered with pentane to afford deep blue needles that were subsequently isolated by filtration and washed with pentane (2 × 10 mL). Yield = 278 mg (75%). **$^{19}F\{^1H\}$ NMR** (282 MHz, CD_2Cl_2): δ -63.6 (s, Ar^F).

All other solvents and reagents are commercial products and were used as received.

NMR spectra were recorded on Bruker DPX-400, AV-400, AV-500, AVIIIHD-500 and AVIII-600 spectrometers at 298 K unless otherwise stated. ^1H NMR spectra recorded in DiFB were referenced using the highest intensity peak of the highest (δ 6.87) frequency fluoroarene multiplet. An internal sealed capillary of 0.25 M $OP(OMe)_3$ in C_6D_6 was used to lock and shim samples for acquisition of NMR data, and additionally act as an internal reference for ^1H and $^{31}P\{^1H\}$ NMR data. Chemical shirts are quoted in ppm and coupling constants in Hz. EPR spectra were acquired on a Bruker EMX spectrometer using a TM_{110} cylindrical mode resonator (ER 4103TM). Samples were cooled by nitrogen gas flow through a standard quartz insert from a nitrogen evaporator with a B-VT 2000 temperature control unit. To limit the dielectric loss arising from the solvent all samples were contained in 2.2 mm i.d. quartz tubes (Wilmad 705-SQ), and the quartz insert was removed for room temperature operation. The reported g-factor is referenced to a DPPH standard (g = 2.0036(3), ref. 3) and all EPR spectra are background subtracted unless otherwise noted. The background was recorded for a sample of [Fc][BAr^F_4] in DiFB under identical conditions giving a featureless spectrum attributed to cavity background (see Figure S18).

ESI-HRMS analyses were recorded on Bruker Maxis Impact instrument.

Microanalyses were performed by Stephen Boyer at London Metropolitan University.

1.2 Preparation of [Pd(P^{t}Bu$_3$)$_2$][PF$_6$] (2a)

A suspension of [Pd(P^{t}Bu$_3$)$_2$] (**1a**, 40 mg, 0.078 mmol) and [Fc][PF$_6$] (24 mg, 0.078 mmol) in DiFB (3 mL) was stirred at room temperature for 2 h. The solution was filtered and the product precipitated with pentane as a Persian blue solid. Yield = 47 mg (92%).

EPR (15 mM in DiFB, 200 K): g_{iso} = 2.316(5), a(^{105}Pd) = 25 mT.

ESI-HRMS (THF, 180 °C, 3 kV): positive ion: 510.2736 *m/z*, [M]$^+$ (calcd. 510.2740 *m/z*).

UV-Vis: (0.15 mM in DiFB, 293 K): λ_{max} (ε) 667 nm (4500 ± 200 M^{-1}·cm^{-1}). First order decrease of this peak is observed at 293 K (k_{obs} = 0.00039 min^{-1}). When ca. 100 equiv. of water was added an increased rate of decomposition was measured (k_{obs} = 0.00076 min^{-1}).

Anal. Calcd for $C_{24}H_{54}F_6P_3Pd$ (656.03 g·mol^{-1}): C, 43.94; H, 8.30; N, 0.00. Found: C, 44.03; H, 8.17; N, 0.00.

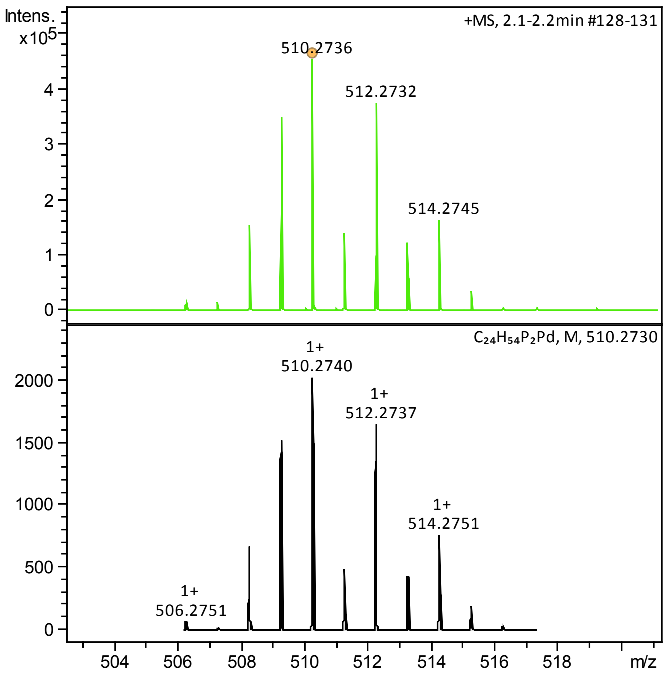

Figure S1. ESI-HRMS of **2a**: observed (above) and calculated (below).

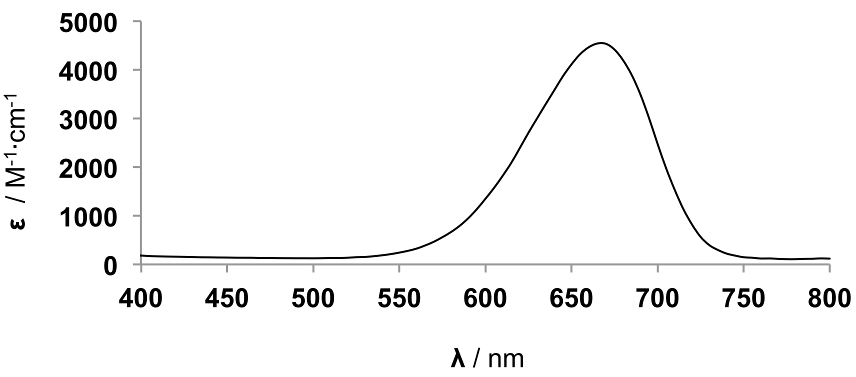

Figure S2. UV-Vis spectrum of **2a** in 1,2-$C_6H_4F_2$ (293 K).

1.3 Preparation of 2,6-bis(decyl)pyridine

To a cooled and stirred solution of lutidine (2.0 mL, 17.3 mmol) in dry THF (60 mL, -78°C) was added ^{n}BuLi (1.6 M in hexanes, 25.6 mL, 41.0 mmol) dropwise. Upon addition, the colourless solution turned bright orange and then red. After 30 minutes stirring at -78˚C, 1-bromononane (7.47 mL, 39 mmol) was added and solution slowly warmed to room temperature over 16 h. The mixture was carefully quenched with water (10 mL) and the organic phase extracted with hexane (3 x 40 mL), washed with water (3 x 10 mL) and dried over $MgSO_4$. Volatiles were removed under vacuum and the residue was purified by silica column chromatography (hexane/ethyl acetate 20:1) to afford the product as a colourless liquid. Yield = 4.10 g (66%).

^{1}H NMR (400 MHz, $CDCl_3$): δ 7.48 (t, $^{3}J_{HH}$ = 7.7, 1H, Py), 6.93 (d, $^{3}J_{HH}$ = 7.7, 2H, Py), 2.74 (app t, J = 7.9, 4H, Py-C$\underline{H_2}$), 1.62 – 1.74 (m, 4H, $PyCH_2C\underline{H_2}$), 1.15 – 1.40 (m, 28H, CH_2), 0.87 (t, $^{3}J_{HH}$ = 6.6, 6H, CH_3).
^{13}C{^{1}H} NMR (126 MHz, $CDCl_3$): δ 162.0 (Py{C}), 136.4 (Py), 119.7 (Py), 38.8 (Py$\underline{C}H_2$), 32.1 ($PyCH_2\underline{C}H_2$), 30.4 (CH_2), 29.8 (CH_2), 29.74 (CH_2), 29.70 (CH_2), 29.6 (CH_2), 29.5 (CH_2), 22.8 (CH_2), 14.3 (CH_3).
ESI-HRMS (CH_3CN, 180 °C, 3 kV): positive ion: 360.3636 *m/z*, $[MH]^+$ (calcd. 360.3625 *m/z*).

1.4 Preparation of [Pt(κ^2_{PC}–P^{t}Bu$_2$CMe$_2$CH$_2$)(P^{t}Bu$_3$)][PF$_6$] (3b)

A solution of [Pt(P^{t}Bu$_3$)$_2$] (**1b**, 75 mg, 0.13 mmol) in DiFB (2 mL) was added dropwise to a mixture of [Fc][PF_6] (87 mg, 0.26 mmol) and 2,6-bis(decyl)pyridine (225 mg, 0.63 mmol) in DiFB (2 mL). After 12 h stirring, the solution was filtered, the volatiles removed under vacuum, and the residue washed with pentane. The crude product was recrystallised from DiFB/pentane to afford the product as yellow crystals. Yield = 86 mg (93%).

^{1}H NMR (600 MHz, CD_2Cl_2): δ 2.75 (br d', $^{3}J_{PH}$ = 7.8, $^{2}J_{PtH}$ = 110, 2H, $PtCH_2$), 1.59 (d, $^{3}J_{PH}$ = 13.1, 6H, P^{t}Bu$_2$C$\underline{Me_2}$CH$_2$), 1.58 (d, $^{3}J_{PH}$ = 14.1, 18H, P$^t\underline{Bu_2}$CMe$_2$CH$_2$), 1.47 (d, $^{3}J_{PH}$ = 13.1, 27H, P$^t\underline{Bu_3}$).
^{13}C{^{1}H} NMR (126 MHz, CD_2Cl_2): δ 56.9 (br d, $^{1}J_{PC}$ = 19, P^{t}Bu$_2\underline{C}$Me$_2$CH$_2$), 42.1 (dd, $^{1}J_{PC}$ = 14, $^{3}J_{PC}$ = 2, P$^t\underline{Bu_3}${C}), 40.4 (dd, $^{1}J_{PC}$ = 10, $^{3}J_{PC}$ = 4, P$^t\underline{Bu_2}$CMe$_2$CH$_2${C}), 32.4 (P$^t\underline{Bu_2}$CMe$_2$CH$_2${CH$_3$}), 32.1 (P$^t\underline{Bu_3}${CH$_3$}), 30.5 (s', $^{3}J_{PtC}$ = 81, P^{t}Bu$_2$C$\underline{Me_2}$CH$_2$), 10.3 (br d', $^{2}J_{PC}$ = 22, $^{1}J_{PtC}$ = 670, $PtCH_2$). Assignments from ^{1}H–^{13}C HSQC and HMBC experiments.
^{31}P{^{1}H} NMR (243 MHz, CD_2Cl_2): δ 59.1 (d', $^{2}J_{PP}$ = 317, $^{1}J_{PtP}$ = 2896, 1P, P^{t}Bu$_3$), 25.2 (d', $^{2}J_{PP}$ = 317, $^{1}J_{PtP}$ = 1916, 1P, P^{t}Bu$_2$CMe$_2$CH$_2$), -144.8 (sept, $^{1}J_{PF}$ = 710, 1P, PF_6). Assignments from ^{1}H–^{31}P HMBC experiment.
^{195}Pt NMR (from ^{1}H–^{195}Pt HMQC experiment, CD_2Cl_2, 225 K): δ –3816.
ESI-HRMS (CH_3CN, 180 °C, 3 kV): positive ion: 598.3284 *m/z*, $[M]^+$ (calcd. 598.3265 *m/z*).
Anal. Calcd for $C_{24}H_{53}F_6P_3Pt$ (743.68 g·mol^{-1}): C, 38.76; H, 7.18; N, 0.00. Found: C, 38.90; H, 7.33; N, 0.00.

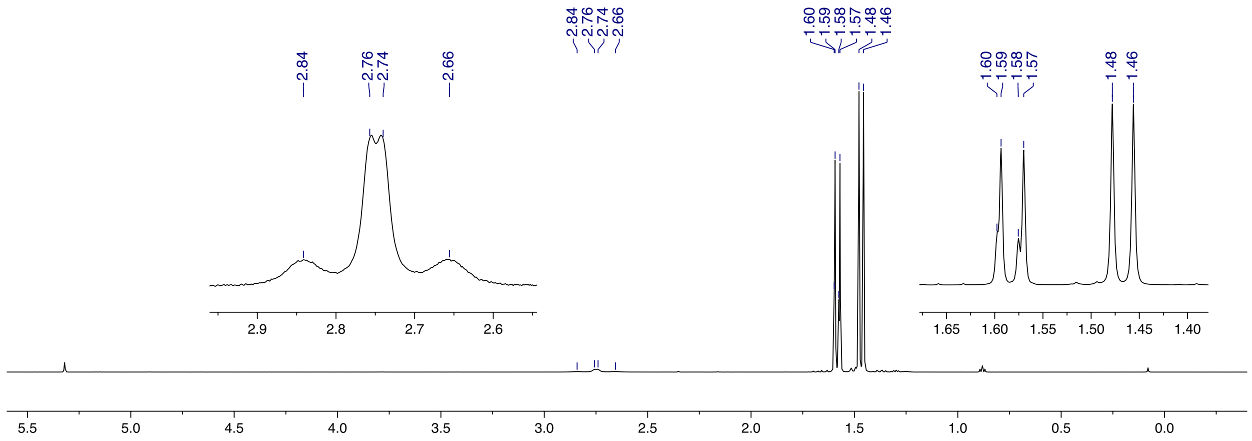

Figure S3. ^{1}H NMR spectrum of **3b** (CD_2Cl_2, 600 MHz, 298 K) – inserts not to scale.

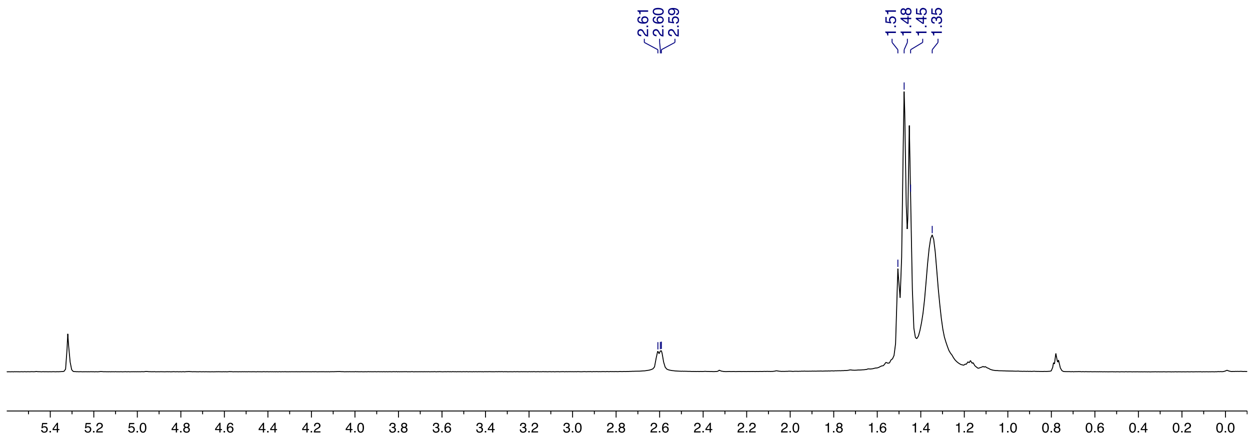

Figure S4. ^{1}H NMR spectrum of **3b** (CD_2Cl_2, 600 MHz, 185 K).

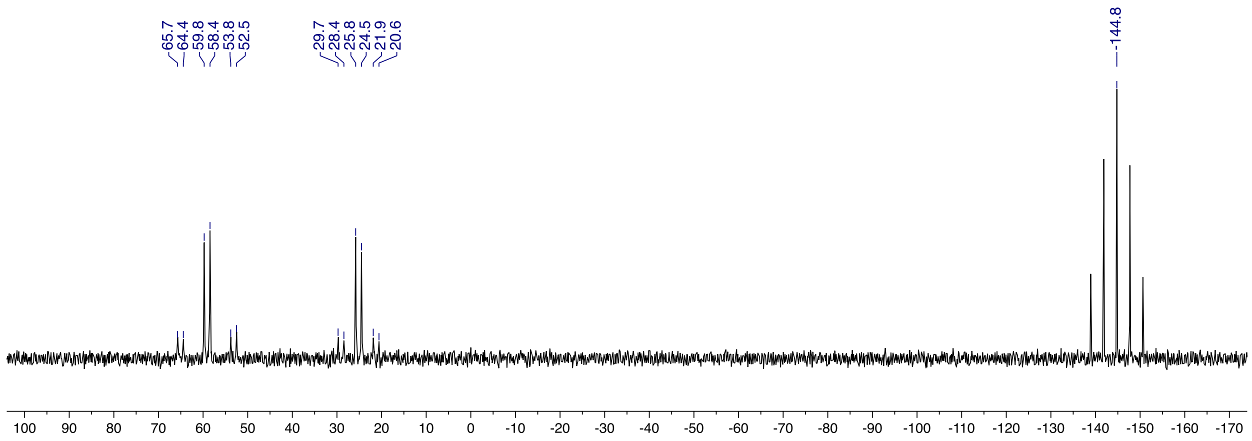

Figure S5. $^{31}P\{^{1}H\}$ NMR spectrum of **3b** (CD_2Cl_2, 243 MHz, 298 K).

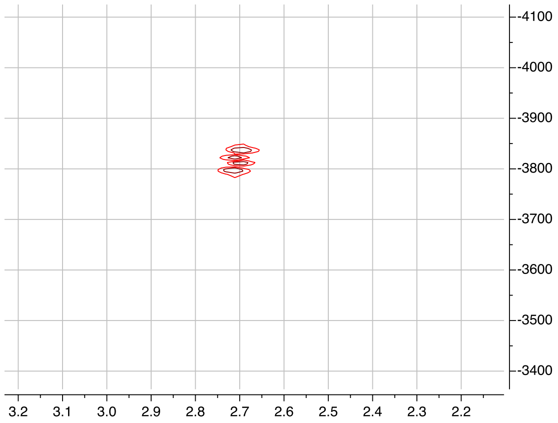

Figure S6. Most relevant section of the ^{1}H–^{195}Pt HMQC spectrum of **3b** (CD_2Cl_2, 225 K).

1.5 Preparation of [Pt(κ^2_{PC}–P^{t}Bu$_2$CMe$_2$CH$_2$)(bipy)][PF$_6$] (6)

A solution of [Pt(κ^2_{PC}–P^{t}Bu$_2$CMe$_2$CH$_2$)(P^{t}Bu$_3$)][PF$_6$] (**3b**, 30 mg, 0.040 mmol) and 2,2'-bipyridine (6.2 mg, 0.040 mmol) in DiFB (2 mL) was stirred for 30 minutes. The volatiles were removed under vacuum, and the residue was washed with pentane. The crude product was recrystallized from DiFB/pentane to afford the product as yellow crystals. Yield = 27 mg (97%).

^{1}H NMR (600 MHz, CD_2Cl_2): δ 8.98 (d, $^3J_{HH}$ = 8.2, 1H, H$^{6'}$), 8.62 (br d', $^3J_{HH}$ = 6.5, 3J_{PtH} = 30, 1H, H^{6}), 8.34 (app d, J = 8, 2H, H$^{3+3'}$), 8.28 (app t, J = 8, 2H, H$^{4+4'}$), 7.70 (t, $^3J_{HH}$ = 6.5, 1H, H^{5}), 7.67 (t, $^3J_{HH}$ = 6.4, 1H, H$^{5'}$), 1.75 (d', $^3J_{PH}$ = 8.2, $^2J_{PtH}$ = 98, 2H, PtCH$_2$), 1.61 (d, $^3J_{PH}$ = 13.6, 18H, P$^t\underline{Bu_2}$CMe$_2$CH$_2$), 1.60 (d, $^3J_{PH}$ = 14.0, 6H, P^{t}Bu$_2$C$\underline{Me_2}$CH$_2$). Assignments aided by NOE experiments.

^{13}C{^{1}H} NMR (151 MHz, CD_2Cl_2): δ 157.1 (C$^{2'}$), 155.5 (C^{2}), 153.7 (C$^{6'}$), 148.8 (C^{6}), 141.4 (C$^{4'}$), 140.5 (C^{4}), 128.9 (C$^{5'}$), 128.2 (C^{5}), 124.4 (C$^{3'}$), 124.1 (C^{3}), 54 (obscured, P^{t}Bu$_2\underline{C}$Me$_2$CH$_2$), 37.4 (d, $^1J_{PC}$ = 16, P$^t\underline{Bu_2}$CMe$_2$CH$_2${C}), 32.7 (s', $^3J_{PtC}$ = 67, P^{t}Bu$_2$C$\underline{Me_2}$CH$_2$), 32.1 (P$^t\underline{Bu_2}$CMe$_2$CH$_2${CH$_3$}), 3.9 (d', $^2J_{PC}$ = 26, $^1J_{PtC}$ = 580, PtCH$_2$). Assignments from ^{1}H–^{13}C HSQC and HMBC experiments.

^{31}P{^{1}H} NMR (202 MHz, CD_2Cl_2): δ -19.9 (s', $^1J_{PtP}$ = 3105, 1P, P^{t}Bu$_2$CMe$_2$CH$_2$), -144.4 (sept, $^1J_{PF}$ = 710, 1P, PF$_6$).

^{195}Pt NMR (from ^{1}H–^{195}Pt HMQC experiment, CD_2Cl_2, 225 K): δ –3788.

ESI-HRMS (CH_3CN, 180 °C, 3 kV): positive ion: 552.2113 *m/z*, [M]$^+$ (calcd. 552.2104 *m/z*).

Anal. Calcd for $C_{24}H_{53}P_2Pt$ (697.54 gmol^{-1}): C, 37.88; H, 4.91; N, 4.02. Found: C, 37.96; H, 4.97; N, 3.87.

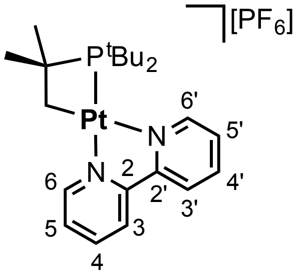

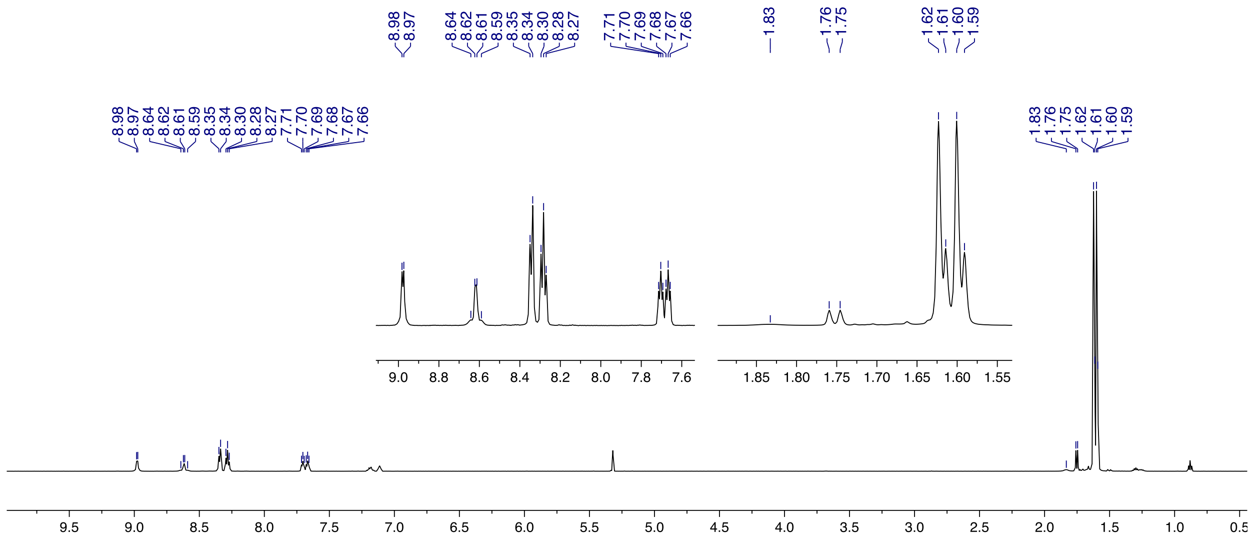

Figure S7. ^{1}H NMR spectrum of **6** (CD_2Cl_2, 600 MHz, 298 K) – inserts not to scale.

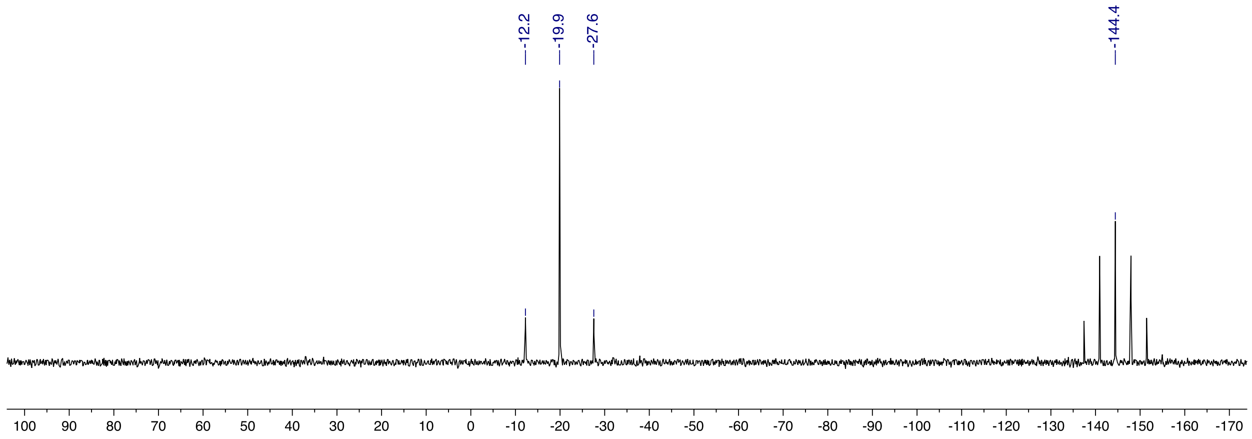

Figure S8. $^{31}P\{^{1}H\}$ NMR spectrum of **6** (CD_2Cl_2, 202 MHz, 298 K).

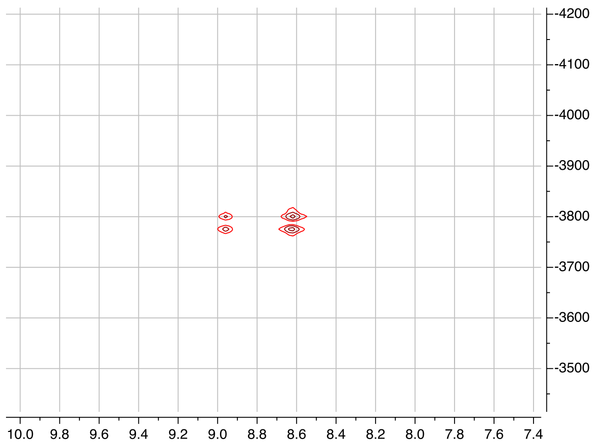

Figure S9. Most relevant section of the ^{1}H–^{195}Pt HMQC spectrum of **6** (CD_2Cl_2, 225 K).

2 NMR scale reaction details

2.1 ^{31}P Chemical shifts in 1,2-$C_6H_4F_2$

Compound	δ_{31P}	Coupling constants / Hz
P^tBu_3	62.2	
$[HP^tBu_3]^+[PF_6]$	ca. 55	
$[Pd(P^tBu_3)_2]$ (**1a**)	84.8	
$[Pt(P^tBu_3)_2]$ (**1b**)	100.1	$^1J_{PtP}$ = 4408
$[Pt(\kappa^2_{PC}–P^tBu_2CMe_2CH_2)(P^tBu_3)]^+[PF_6]$ (**3b**)	24.3, 59.0	$^1J_{PtP}$ = 1916, 2898; $^2J_{PP}$ = 317
$[Pt(P^tBu_3)_2H]^+[PF_6]$ (**4**)	86.3	$^1J_{PtP}$ = 2621
$[Pt(\kappa^2_{PC}–P^tBu_2CMe_2CH_2)(bipy)]^+[PF_6]$ (**6**)	-20.1	$^1J_{PtP}$ = 3105

All relative to an internal sealed capillary containing $OP(OMe)_3/C_6D_6$
[δ_{31P} 3.1, δ_{1H} 3.36 ($^3J_{PH}$ = 11.0)]

2.2 General conditions

Reactions were carried in 5 mm J. Young's valve NMR tubes using 0.015 mmol complex (i.e. 7.7 mg **1a**, 9.0 mg **1b**, 9.9 mg **2a**, 11.2 mg **3b**) in DiFB (0.50 mL) solvent and an internal capillary containing 60 µL of a 0.25 M solution of trimethylphosphate in C_6D_6. Reactions were monitored by ^1H and ^{31}P NMR spectroscopy.

2.3 Reactions of $[Pd(P^tBu_3)_2]$ (1a)

2.3.1 **1a** @ 293 K

No change apparent after 24 h.

2.3.2 **1a** + 1 equiv. $[Fc][PF_6]$ @ 293 K

No diamagnetic species observed in the $^{31}P\{^1H\}$ NMR spectrum after 15 min. The ^1H NMR spectrum showed the presence of Fc (δ_{1H} 4.14).

2.3.3 **1a** + 5 equiv. 2,6-bis(decyl)pyridine @ 293 K

No reaction apparent after 24 h.

2.3.4 **1a** + 2 equiv. $[Fc][PF_6]$ + 5 equiv. 2,6-bis(decyl)pyridine @ 293 K

Slow formation of a new organometallic species characterised by two doublets in the $^{31}P\{^1H\}$ NMR spectrum in a 1:1 ratio (δ_{31P} 57.0, -1.3; $^2J_{PP}$ = 316 Hz). After 72 h an approximate conversion of 30% was determined using the internal reference (Figure S10).

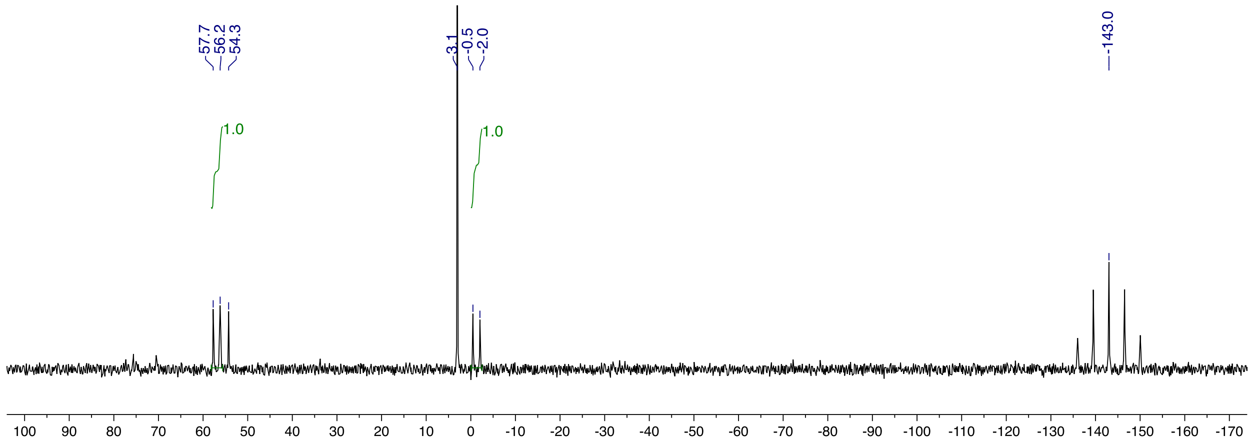

Figure S10. $^{31}P\{^{1}H\}$ NMR spectrum (DiFB, 202 MHz, 298 K).

2.4 Reactions of [Pt(P^{t}Bu$_3$)$_2$] (1b)

2.4.1 **1b** @ 293 K

No change apparent after 24 h.

2.4.2 **1b** @ 353 K

No change apparent after 24 h.

2.4.3 **1b** + 1 equiv. [Fc][PF_6] @ 293 K

1:1 mixture of **3b** and **4** formed within 15 min by ^{31}P NMR spectroscopy. Integration against the internal standard indicates complete conversion. The ^{1}H NMR spectrum showed the presence of Fc (δ_{1H} 4.01).

2.4.4 **1b** + 5 equiv. 2,6-bis(decyl)pyridine @ 293 K

No reaction apparent after 24 h.

2.4.5 **1b** + 2 equiv. [Fc][PF_6] + 5 equiv. 2,6-bis(decyl)pyridine @ 293 K

Integration against the internal standard indicated complete conversion to **3b** within 15 min. The ^{1}H NMR spectrum showed the presence of Fc (δ_{1H} 4.63).

2.4.6 **1b** + 1 equiv. [Fc][PF_6] + 1 equiv. 2,2'-bipyridine @ 293 K

Integration against the internal standard indicated formation of a mixture comprised of **1b**, **6**, P^{t}Bu$_3$ and [HP^{t}Bu$_3$]$^+$ (~1:1:0.3:0.7) by ^{31}P NMR spectroscopy after 15 min. The ^{1}H NMR spectrum showed the presence of Fc (δ_{1H} 3.97).

2.4.7 **1b** + 2 equiv. [Fc][PF_6] + 1 equiv. 2,2'-bipyridine @ 293 K

Integration against the internal standard indicated complete conversion to a ~1:1 mixture comprised of **6** and [HP^{t}Bu$_3$]$^+$ by ^{31}P NMR spectroscopy after 15 min. The ^{1}H NMR spectrum showed the presence of Fc (δ_{1H} 3.97).

2.5 Reactions of [Pd(P^{t}Bu$_3$)$_2$][PF$_6$] (2a)

2.5.1 **2a** @ 293 K

No new diamagnetic species observed in either the ^{1}H or ^{31}P{^{1}H} NMR spectrum after 24 h.

2.6 Reactions of [Pt(κ^2_{PC}–P^{t}Bu$_2$CMe$_2$CH$_2$)(P^{t}Bu$_3$)][PF$_6$] (3b)

2.6.1 **3b** @ 293 K

No change apparent after 24 h.

2.6.2 **3b** + H_2 (1 atm) @ 293 K

Complete conversion to **4** after 1 h shaking apparent by ^{1}H and ^{31}P NMR spectroscopy. After freeze-pump-thaw-degassing and placing under an argon atmosphere, no additional reaction was apparent after 15 min (δ_{1H} –36.30, $^2J_{PH}$ = 8.6, $^1J_{PtH}$ = 2590 Hz; δ_{31P} 86.3, $^1J_{PtP}$ = 2621 Hz; Figure S11 and S12).

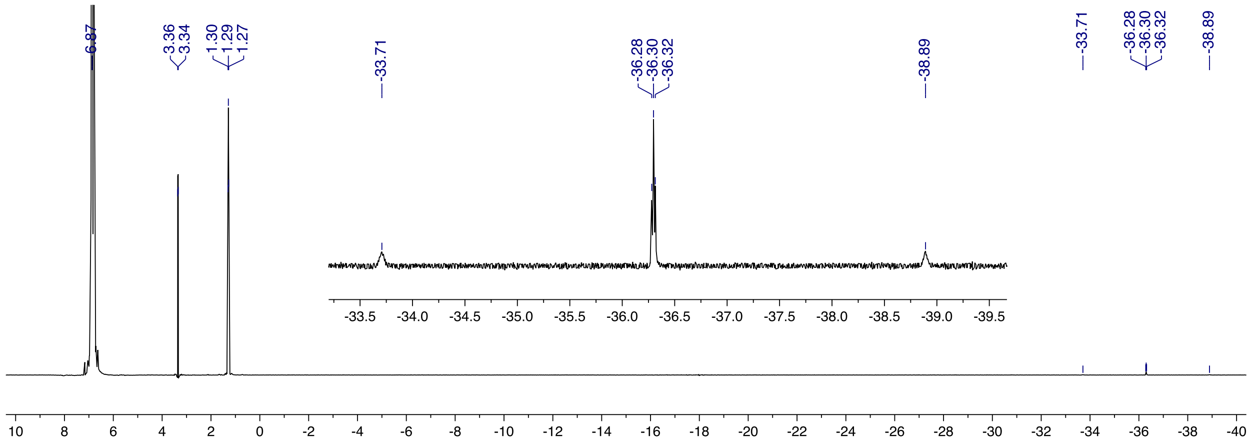

Figure S11. ^{1}H NMR spectrum of **4** (DiFB, 500 MHz, 298 K).

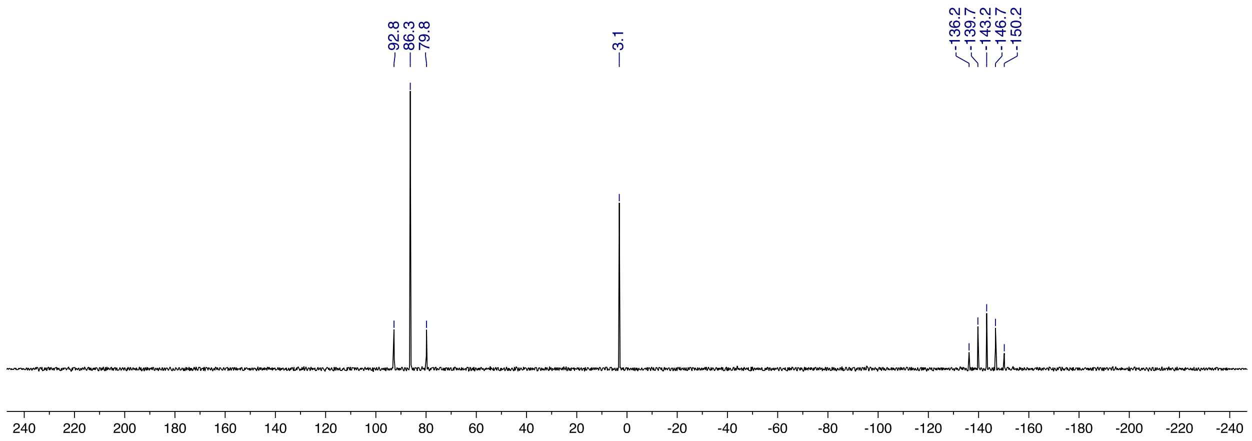

Figure S12. ^{31}P{^{1}H} NMR spectrum of **4** (DiFB, 202 MHz, 298 K).

To the mixture described above was added 5 equiv. of 2,6-bis(decyl)pyridine, resulting in deprotonation of **4** and formation of an equilibrium mixture of **1b** and **4** in a 1:7 ratio (by ^{31}P NMR

spectroscopy) after 15 min (ratio unchanged after an additional 45 min).

2.6.3 **3b** + 1 equiv. 2,2'-bipyridine @ 293 K

Quantitative formation of **6** with concomitant liberation of free P^tBu_3 was observed within 15 min by ^{31}P NMR spectroscopy.

3 Electrochemistry

3.1 General methods

Cyclic voltammetry (CV) experiments were carried out in an inert atmosphere glovebox under argon out using a CHI 760 C potentiostat (CH Instruments, Inc.) in a typical 3-electrode set-up where a glassy carbon substrate, platinum mesh and silver wire were used as the working (WE), counter (CE) and reference electrode (RE), respectively. All potentials are calibrated to the ferrocene/ferrocenium (Fc/$[Fc]^+$) redox couple which was used as an internal standard.

The half-wave potentials, $E_{1/2}$ were determined from:

$$E_{1/2} = (E_p^{red} + E_p^{ox}) / 2$$

where E_p^{red} and E_p^{ox} are the reduction and oxidation peak potentials, respectively.

3.2 Fc/$[Fc]^+$ redox couple in 1,2-$C_6H_4F_2$ / 0.2 M [$^nBu_4N$][PF_6]

Figure S13 shows typical CVs for the oxidation of Fc at different scan rates. Linear dependence of the voltammogram peak current, i_p to square root of the potential sweep scan rate, $v^{1/2}$ indicates a diffusion-controlled process.

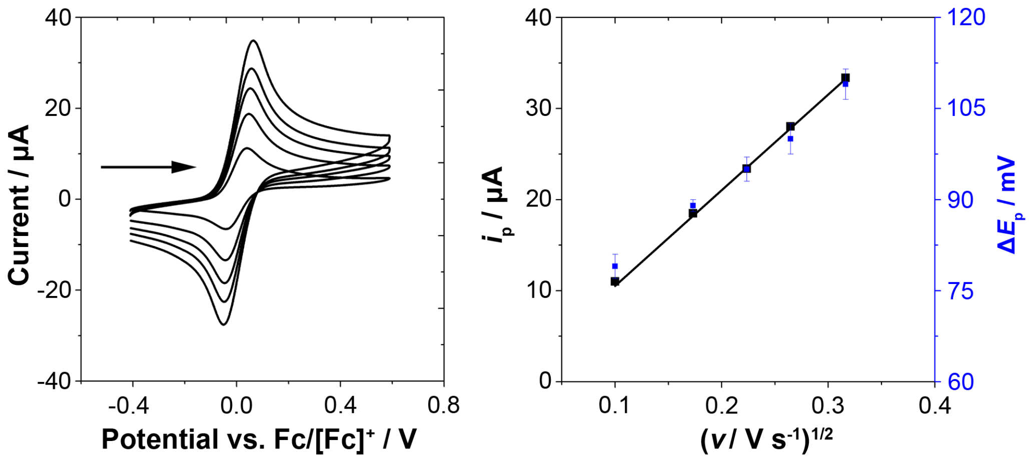

Figure S13. (a) CVs for the oxidation of Fc in 1,2-$C_6H_4F_2$ (2 mM; 0.2 M [$^nBu_4N$][PF_6] electrolyte; scan rates = 10, 30, 50, 70 and 100 mV s^{-1}); (b) Plot of i_p and ΔE_p versus $v^{1/2}$.

The peak-to-peak potential separation, ΔE_p is 108 mV at ν = 100 mV s^{-1} which deviates from the expected value of 60 mV (reversible) for the Fc/[Fc]$^+$ redox couple.[4] This is reasonably attributed to the high internal resistance of the solution arising from incomplete ionic dissociation resulting in ohmic resistance of ~ 1 KΩ. Furthermore, i_p^{red} / i_p^{ox} ~ 0.99 is characteristic of a chemically reversible process.

3.3 Reduction of isolated 2a

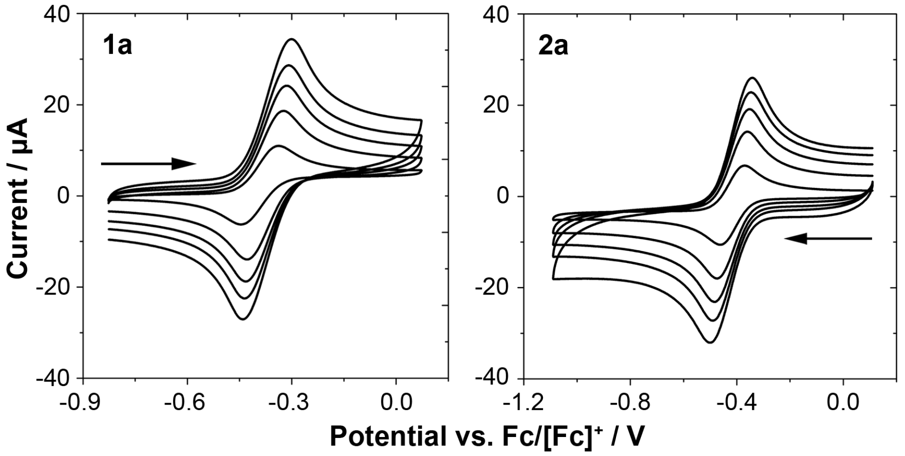

Figure S14. CVs for the oxidation of **1a** and reduction of **2a** in 1,2-$C_6H_4F_2$ (2 mM [Pd]; 0.2 M [^{n}Bu$_4$N][PF$_6$] electrolyte; scan rates = 10, 30, 50, 70 and 100 mV s^{-1}).

3.4 Reduction of isolated 3b[5] and 6

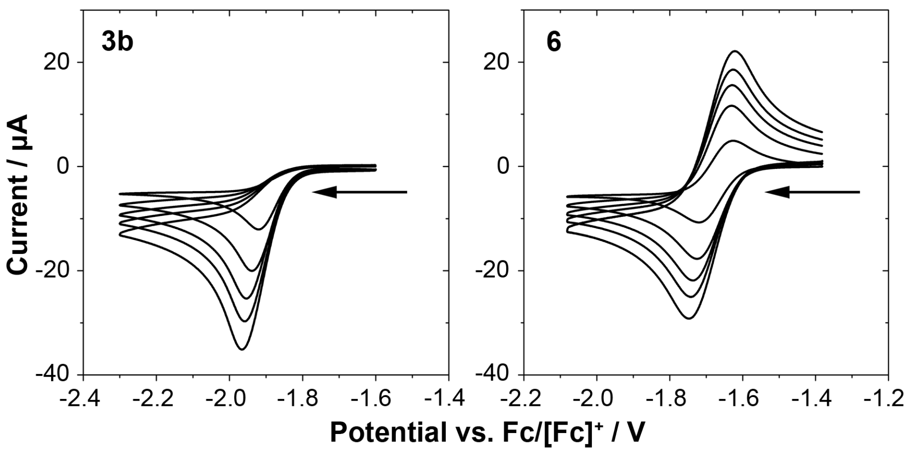

Figure S15. CVs for the reduction of **3b** and **6** in 1,2-$C_6H_4F_2$ (2 mM [Pt]$^+$; 0.2 M [^{n}Bu$_4$N][PF$_6$] electrolyte; glassy carbon working electrode, Pt counter electrode and Ag wire reference electrode; scan rates = 10, 30, 50, 70 and 100 mV s^{-1}).

3.5 Additional details

Diffusion coefficients, *D* were determined using the Randle-Sevcik[4] equation:

$$i_p = 2.69\times10^5\, n^{3/2} A C D^{1/2} \nu^{1/2}$$

where *n* is the number of electrons transferred per redox event, *A* is the electrode area and *C* is the concentration.

Table S1. Calculated diffusion coefficients from Randle-Sevcik analysis ($1,2\text{-}C_6H_4F_2$, 0.2 M $[^nBu_4N][PF_6]$ electrolyte)

Compound	R^2 (fit)	D / 10^{-6} cm^2 s^{-1}
**Fc**	0.99995	7.6
**1a**	0.99964	6.9
**1b**	0.99938	5.3
**2a**	0.99836	5.9
**3b**	0.99780	7.6
**6**	0.99950	5.9

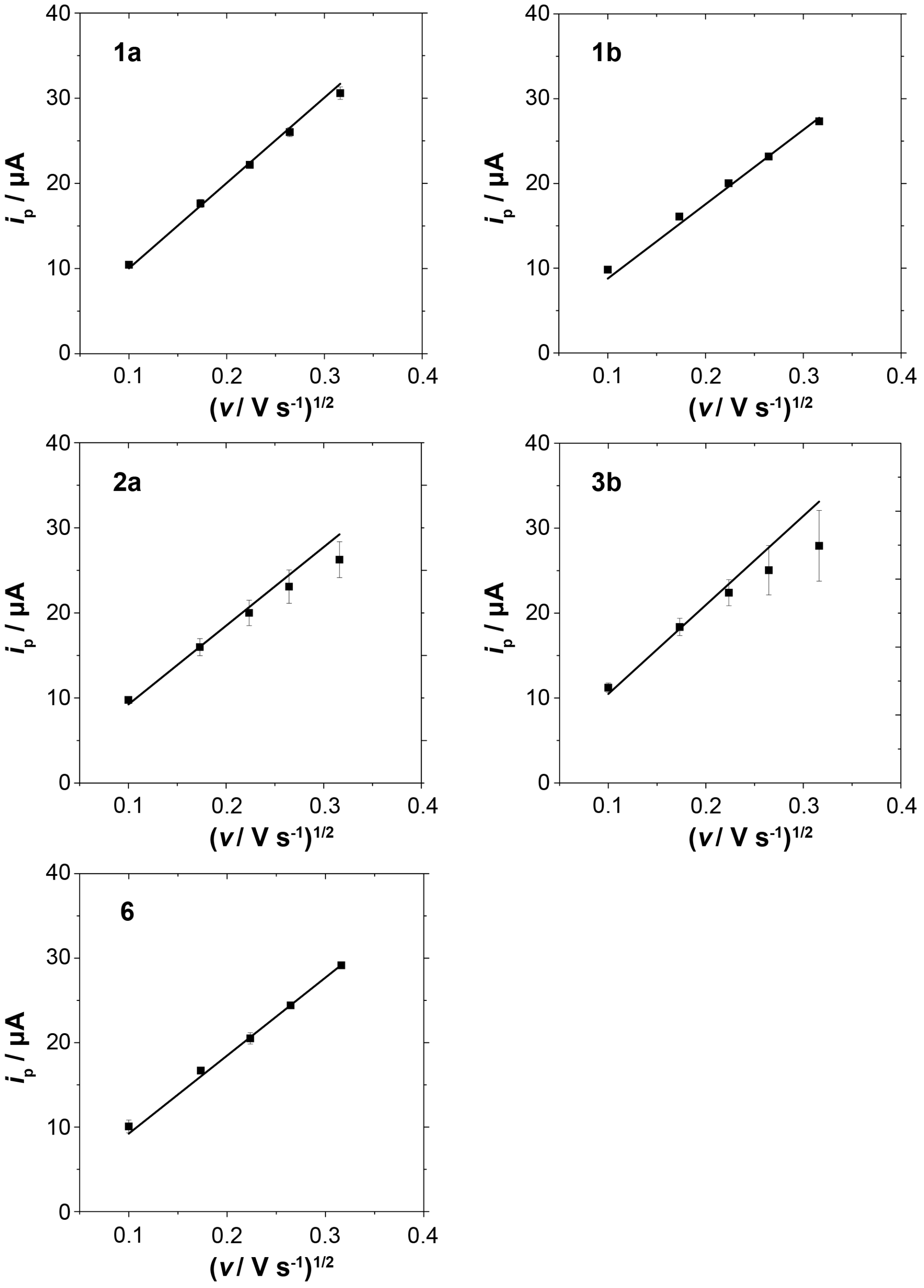

Figure S16. Plots of i_p versus $v^{1/2}$ for **1a**, **1b**, **2a**, **3b** and **6**.

4 EPR Spectroscopy

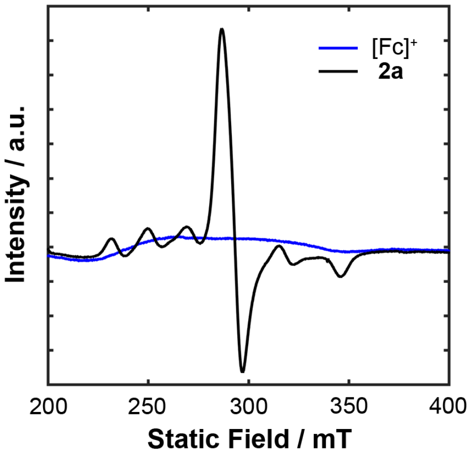

Figure S17. EPR spectra of **2a** and [Fc][BAr$^F_4$] (1,2-$C_6H_4F_2$ glass, 200 K, a.u. = arbitrary units) without baseline correction.

5 Crystallography

Full crystallographic details including solution, refinement and disorder modelling procedures are document in CIF format and have been deposited with the Cambridge Crystallographic Data Centre under CCDC 1440602 (**2a**), 1440603 (**3b**) 1440604 (**6**, C_2/c) and 1440605 (**6**, $P2_1/c$). These data can be obtained free of charge from The Cambridge Crystallographic Data Centre via www.ccdc.cam.ac.uk/data_request/cif. Notably two different solid-state structures for **6** were obtained. Both sets of data were collected from crystals grown from the same solvent, but the samples crystallised in different space groups. In the $P2_1/c$ structure the cation and anion are extensively disordered over two sites (Figure S18, left), however, only a small degree of disorder is observed in the C_2/c structure (Figure S18, right). In the latter case, only disorder of the (heavy) platinum atom was modelled due to the very low occupancy of the minor component (5%). The data presented in Figure 3 is from the C_2/c structure.

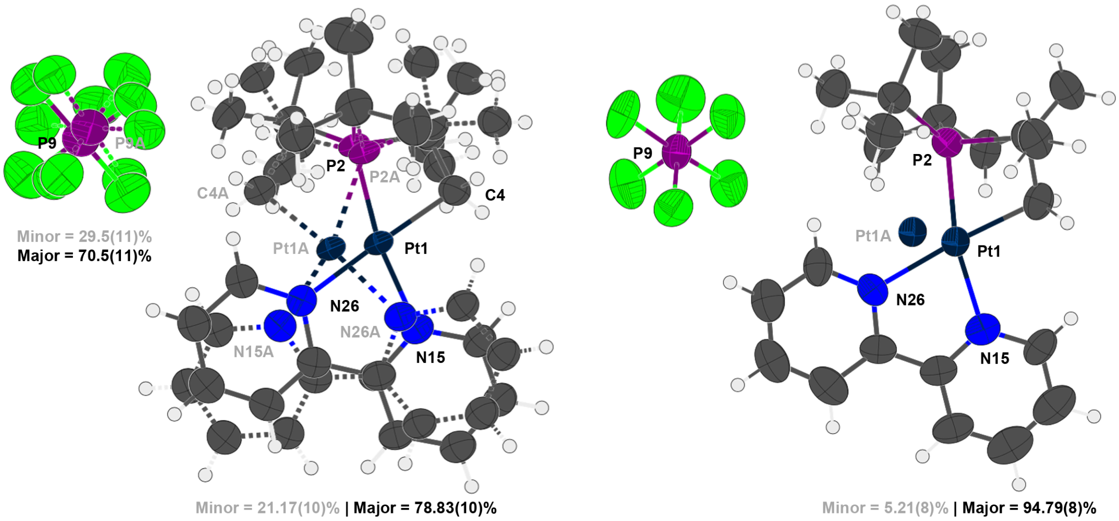

Figure S18. Solid-state structures of **6**: $P2_1/c$ (left, CCDC 1440605) and C_2/c (right, CCDC 1440604). Thermal ellipsoids drawn at 50% probability level. Minor disordered components labelled in grey and bearing A as a suffix. Selected data: $P2_1/c$, Pt1–Pt1A, 1.050(2) Å; C_2/c, Pt1–Pt1A, 0.995(7) Å.

6 References and notes

[1] W. E. Buschmann, J. S. Miller, K. Bowman-James, C. N. Miller, *Inorg. Synth.* **2002**, *33*, 83–91.

[2] I. Chávez, A. Alvarez-Carena, E. Molins, A. Roig, W. Maniukiewicz, A. Arancibia, V. Arancibia, H. Brand, J. M. Manríquez, *J. Organomet. Chem.* **2000**, *601*, 126–132.

[3] J. Krzystek, A. Sienkiewicz, L. Pardi, L. C. Brunel, *J. Magn. Reson.*, **1997**, *125*, 207–211.

[4] A. J. Bard, L. R. Faulkner. *Electrochemical Methods: Fundamentals and Applications* (2nd Ed.), John Wiley and Sons, New York, **2001**.

[5] No oxidation of **3b** is observed on scanning the potential up to +1.0 V vs Fc/[Fc]$^+$.
